# The Balanced Opioid Initiative: protocol for a clustered, sequential, multiple-assignment randomized trial to construct an adaptive implementation strategy to improve guideline-concordant opioid prescribing in primary care

**DOI:** 10.1186/s13012-020-00990-4

**Published:** 2020-04-25

**Authors:** Andrew Quanbeck, Daniel Almirall, Nora Jacobson, Randall T. Brown, Jillian K. Landeck, Lynn Madden, Andrew Cohen, Brienna M. F. Deyo, James Robinson, Roberta A. Johnson, Nicholas Schumacher

**Affiliations:** 1 Department of Family Medicine and Community Health, University of Wisconsin–Madison, 800 University Bay Drive, Suite 210, Madison, WI 53705-2278 USA; 2 Department of Statistics and Institute for Social Research, University of Michigan, 2448 Institute for Social Research, 426 Thompson St., Ann Arbor, MI 48104-2321 USA; 3 Institute for Clinical and Translational Research and School of Nursing, University of Wisconsin, Madison, 5130 Signe Skott Cooper Hall, 701 Highland Ave, Madison, WI 53705-2202 USA; 4 Department of Family Medicine and Community Health, University of Wisconsin–Madison, 1100 Delaplaine Ct, Madison, WI 53705-1840 USA; 5 APT Foundation, 1 Long Wharf Drive, Suite 321, New Haven, CT 06511-5991 USA; 6 Bellin Health Systems, Inc., 744 S. Webster Ave, Green Bay, WI 54305 USA; 7 Forward Data Analytic Services, LLC, 6700 Cross Country Road, Verona, WI 53593 USA

**Keywords:** Clinical guideline adoption, Adaptive implementation strategy, Opioid prescribing, Primary care, Educational meetings, Audit and feedback, Practice facilitation, Prescriber peer consulting, Multi-phase optimization strategy, Clustered SMART

## Abstract

**Background:**

Rates of opioid prescribing tripled in the USA between 1999 and 2015 and were associated with significant increases in opioid misuse and overdose death. Roughly half of all opioids are prescribed in primary care. Although clinical guidelines describe recommended opioid prescribing practices, implementing these guidelines in a way that balances safety and effectiveness vs. risk remains a challenge. The literature offers little help about which implementation strategies work best in different clinical settings or how strategies could be tailored to optimize their effectiveness in different contexts. Systems consultation consists of (1) educational/engagement meetings with audit and feedback reports, (2) practice facilitation, and (3) prescriber peer consulting. The study is designed to discover the most cost-effective sequence and combination of strategies for improving opioid prescribing practices in diverse primary care clinics.

**Methods/design:**

The study is a hybrid type 3 clustered, sequential, multiple-assignment randomized trial (SMART) that randomizes clinics from two health systems at two points, months 3 and 9, of a 21-month intervention. Clinics are provided one of four sequences of implementation strategies: a condition consisting of educational/engagement meetings and audit and feedback alone (EM/AF), EM/AF plus practice facilitation (PF), EM/AF + prescriber peer consulting (PPC), and EM/AF + PF + PPC. The study’s primary outcome is morphine-milligram equivalent (MME) dose by prescribing clinicians within clinics. The study’s primary aim is the comparison of EM/AF + PF + PPC versus EM/AF alone on change in MME from month 3 to month 21. The secondary aim is to derive cost estimates for each of the four sequences and compare them. The exploratory aim is to examine four tailoring variables that can be used to construct an adaptive implementation strategy to meet the needs of different primary care clinics.

**Discussion:**

Systems consultation is a practical blend of implementation strategies used in this case to improve opioid prescribing practices in primary care. The blend offers a range of strategies in sequences from minimally to substantially intensive. The results of this study promise to help us understand how to cost effectively improve the implementation of evidence-based practices.

**Trial registration:**

NCT 04044521 (ClinicalTrials.gov). Registered 05 August 2019.

Contributions to the literature

- The volume of prescription opioids entering the US society through the healthcare system has precipitated a long-term public health crisis. Effective implementation strategies are needed to optimize the application of opioid prescribing and monitoring practices in primary care settings.
- Although experts recognize that implementation strategies should be tailored to specific contexts, the literature offers little guidance on how to do it.
- This study aims to provide guidance on how best to sequence and combine implementation strategies for opioid prescribing to meet the needs of different primary care clinics.

## Background

Opioids are commonly prescribed in primary care to relieve chronic non-cancer pain. Although opioids are indicated for some patients, no scientifically rigorous studies with adequate periods of observation are available to optimally guide patient selection and monitoring practices [1]. Accompanying burdens have become clear and widespread. By 2017, drug overdose was the leading cause of accidental death in the USA. Although the volume of opioids prescribed in the USA declined each year from 2010 to 2015, about three times more opioids were prescribed per person in 2015 as in 1999, and prescribing rates still vary greatly, with the highest prescribing counties prescribing six times more opioids per person than the lowest prescribing counties [2]. About half of opioid prescriptions are written in primary care [1, 3]. Clinical guidelines for opioid prescribing in primary care have been advanced, most notably those issued by the US Centers for Disease Control and Prevention in 2016 [1]. Clinical guidelines provide expert consensus around a few basic ideas: (1) Physicians should discuss the risks and benefits of opioid therapy with patients by reviewing and signing formal treatment agreements before initiating the first opioid dose and throughout treatment [1]. (2) Clinicians should avoid prescribing opioids in doses higher than 90–100 morphine milligram equivalent (MME) daily, since evidence shows that patients with a dose of 100 MME or greater are 11 times more likely to die from overdose than patients taking doses less than 20 MME [4–6]. (3) Patients at increased risk for misuse (i.e., those with mental health or substance use disorders) are more likely to receive opioid prescriptions and higher doses; thus, screening for mental health and substance use disorders should be in place [4, 7–9]. (4) Opioid-benzodiazepine co-prescribing in any combination of doses should be avoided to reduce the risk of overdose [10]. (5) Monitoring via urine drug testing should be instituted to ensure appropriate use of opioid medications [11].

The CDC’s guideline and widespread media attention to the problem of opioid prescribing have produced gains. In July 2019, the CDC published provisional data showing a 5.1% decrease in drug overdose deaths (most of which are caused by opioids) in 2018 compared with 2017 [12]. Despite this positive news, the authors of the CDC guideline published in June 2019 a warning about misapplications of the guideline, such as reducing opioids used to treat cancer pain, rapid tapering of long-term opioid users, sudden discontinuation of opioid therapy, and prescribers’ dismissing patients taking opioids from their practices [13]. In October 2019, the US Department of Health and Human Services published a guide warning clinicians against abruptly discontinuing or tapering patients from long-term opioid use [14, 15]. Clearly, guideline-concordant care is complicated and remains a challenge in 2020.

More broadly, the US healthcare system is notoriously slow to adopt established guidelines or other evidence-based practices (EBPs), regardless of the condition [16]. Lau et al. conducted a 2015 review [17] of 91 studies aimed at determining the effectiveness of strategies for the implementation of complex interventions in primary care settings. The most commonly used strategies were targeted at individual providers, generally demonstrating modest effects, with considerable variability in effectiveness between studies. The authors found little use of implementation strategies targeted at organizations (e.g., the clinic) or a wider context (e.g., health systems). Finally, the review found very limited data on the costs and cost-effectiveness of different implementation strategies. The authors concluded that the literature remains unclear about which implementation strategies should be used under what conditions, and that future research should study implementation strategies targeted at levels broader than individual providers.

The current study (R01DA047279)—The Balanced Opioid Initiative—builds on preliminary research conducted in a pilot study funded by a 3-year R34 clinical trial planning grant from the National Institute on Drug Abuse (R34-DA036720), the results of which were published in 2018 [18]. The mixed-methods pilot study tested the feasibility and acceptability of *systems consultation*, which is a theoretically and empirically grounded [18], multi-component implementation intervention [19, 20].

In the pilot, systems consultation consisted of a bundle of three discrete implementation strategies provided contemporaneously to four primary care clinics over the course of a 6-month period: (1) audit and feedback (henceforth AF, providing baseline performance feedback to clinics and ongoing information that points to progress and opportunities for improvement), (2) practice facilitation (PF, help in tailoring guidelines to specific clinical contexts and processes through workflow assessment and workflow changes), and (3) prescriber peer consulting (PPC, in which a respected physician expert in opioid management provides advice on how to improve clinical practice and address issues with more challenging cases). Four clinics received systems consultation, and four clinics served as controls; see [18] for details. During the 6-month intervention period, the systems consultation implementation team (consisting of a physician expert and a practice facilitator) met with clinic staff in monthly site visits, videoconferences, or teleconferences. The systems consultation strategy generally proved to be feasible and acceptable: Attendance by clinic staff at intervention meetings was 83%, and more than 80% of intervention participants agreed or strongly agreed with the statements, “I am more familiar with guidelines for safe opioid prescribing” and “My clinic’s workflow for opioid prescribing is easier.” In addition, systems consultation appeared to be effective: Compared with control clinics, intervention clinics reduced average morphine-milligram equivalent (MME) dose for patients on long-term opioid therapy by 19.7% over 12 months.

A qualitative formative evaluation conducted during the pilot study [21] yielded a number of observations suggesting modifications to the systems consultation intervention. We used participant observation, focus groups, interviews, and activity tracking to collect data used to follow implementation over time and explore what worked well and what required modification. Synthesizing these data and discussing them among research team members informed plans for the current study as follows. (1) The absence of a dedicated informational session at the health system level—i.e., a meeting designed to inform and engage participants on a wide scale—was a missed opportunity to (a) galvanize common support and engage all primary care clinics and health system leaders around the importance of guideline-concordant prescribing, and (b) introduce audit and feedback (a health-system-level strategy) to clinicians and frontline staff. In addition, the absence of an educational and engagement meeting also meant that (c) initial clinic visits often involved repeatedly explaining the rationale for systems consultation, leading to greater cost to and time burden on the implementation team. (2) Clinicians and frontline staff were better prepared to work on the activities that are part of PF (e.g., workflow changes) after receiving clinic-level performance data supplied by AF reports, which summarized baseline performance and provided a vehicle for ongoing progress monitoring. (3) We developed a team-based implementation and engagement model using both a physician expert and a practice facilitator because it quickly became clear that assigning sole responsibility to the physician expert for advising, communicating, and coordinating with change teams (at the clinic) was overly burdensome and not scalable. We added a practice facilitator to support the physician expert at all monthly PF sessions and facilitate follow-up communications over the course of 6 months. However, it appeared that these roles (physician expert and facilitator) could feasibly be sequenced into separate implementation stages with different foci and different targets of action (i.e., focusing initially on support staff, and later—if needed—on both support staff and prescribers). (4) In three of four pilot clinics, we learned that clinicians and frontline staff charged with implementing new practices often lacked the time and skills to conduct organizational change projects and benefitted from monthly follow-up; yet in one of the four clinics, a single site visit led by a respected physician peer consultant was all that was needed to enact change, lessening the value of subsequent follow-up engagements. The case of this particular clinic gave us reason to believe that an effective educational and engagement meeting might be sufficient to effect positive change in some clinics, without the need for more intensive implementation support. (5) We observed that different clinics prioritized different focal problems that may be better addressed using different combinations of the implementation strategies. For example, regular urine drug screening can be addressed by PF; opioid-benzodiazepine co-prescribing can be addressed by PPC. (6) Candidate determinants of the success of different implementation strategies were identified across multiple levels: the existence of a related health system policy (at health system level), the overall size of the clinic panel and experience doing quality improvement work (at the clinic level), and the number of high-dose opioid patients and “inherited” opioid patients (at the prescriber level).

**Fig. 1:**
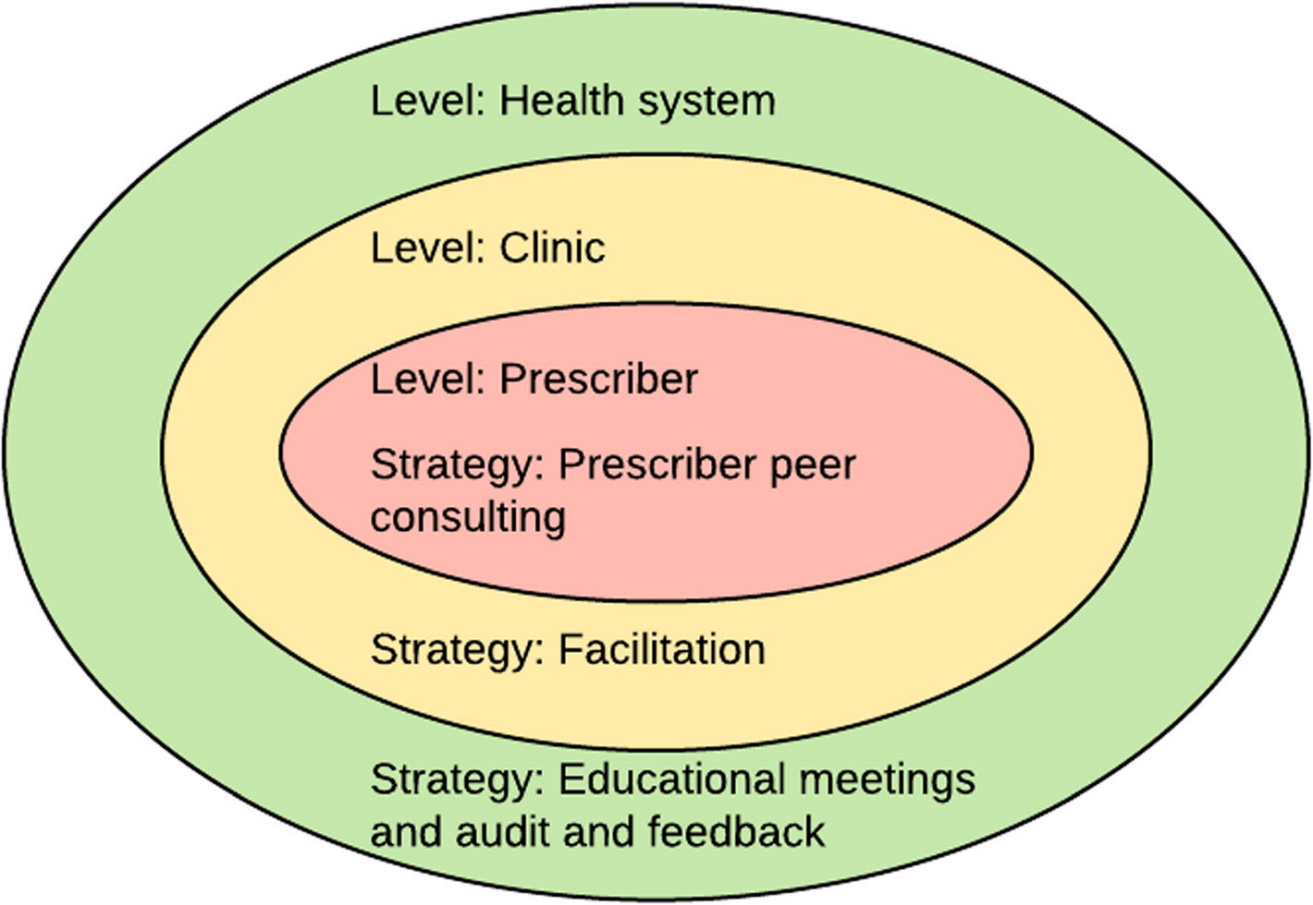
Theoretical and empirical framework. Sources: Ferlie and Shortell [22]; Powell [19]

These insights suggested a set of modifications to systems consultation that informed the design of the current study. We added an educational/engagement meeting (EM, with the option of receiving continuing medical education credits) as a complementary health system strategy alongside AF. Our experience with the pilot suggested the need for a sequenced approach to the strategies within systems consultation, whereby potentially more intensive strategies are offered only after providing less intensive strategies. In the current study, we operationalize this idea by first providing EM/AF to all clinics in a first stage of implementation, then considering whether to augment EM/AF with PF in a second stage of implementation, and then considering whether to augment with PPC in a third stage of implementation. This approach—considering whether to progress from broad organizational support via EM/AF (the least intensive set of strategies) to support for individual prescribers via PPC (the most intensive)—aligns well with existing multi-level frameworks guiding implementation [19, 22] (see Fig. 1). Finally, our experience also suggested the need to consider a tailored approach to delivering the strategies within systems consultation [23, 24], whereby different clinics may be provided PF depending on their needs following EM/AF, and different clinics may be provided PPC depending on their needs following prior intervention (e.g., EM/AF followed by PF).

This combination of a sequenced and tailored approach to systems consultation is known as an *adaptive implementation strategy* [25–30]. However, two sets of challenges prevent us from evaluating or recommending such an approach to addressing opioid prescribing in primary care using the results of our pilot alone. First, currently there is no empirical support for the effectiveness of the PF and/or PPC strategies (singly or jointly) following EM/AF. Second, there is currently no empirical support, nor guidance, on whether or how—i.e., based on which measures—to tailor PF or PPC to the needs of different clinics. This study aims to address these important gaps in the literature.

### Overall goal

The ultimate goal of this optimization study is a proposal for an adaptive implementation strategy, *adaptive systems consultation*, that provides clear guidance to implementation decision-makers about when and for which clinics to provide PF and PPC in the context of EM/AF for the purpose of improving opioid prescribing in primary care settings. We will do this by addressing the following specific aims. Taken together, these aims test the effectiveness of the PF and PPC strategies and seek to improve our understanding of how best to dynamically tailor PF and PPC to the needs of the primary care clinics.

### Primary aim

The study’s primary aim is to compare (1) clinics offered the most intensive sequence of strategies (EM/AF + PF + PPC) vs. (2) clinics offered the least intensive strategy (EM/AF alone) on change in clinic-level, average morphine-milligram equivalent dose (the primary outcome) from the end of month 3 (the point at which a clinic may be randomized to PF) to the end of month 21 (end of intervention).

### Secondary aim

The study will also estimate the cost of delivering four different sequences and combinations of strategies (EM/AF, EM/AF + PF, EM/AF + PPC, and EM/AF + PF + PPC), including the incremental cost effectiveness of adding facilitation and prescriber peer consulting. Results will help decision-makers weigh the costs and effects of using different sequences of implementation strategies.

### Exploratory analyses

The study will conduct exploratory analyses to understand contextual factors that influence the effectiveness of the different sequences of implementation strategies. We conjecture that two factors are useful to consider in deciding at the end of month 3 whether to add PF: (a) a clinic’s experience of doing quality improvement and (b) size of clinic. At the end of month 9, we posit that those two factors plus two others are useful to consider in deciding whether to add PPC: (c) whether PF was offered at the end of month 3 and (d) number or percent of high-dose opioid patients at the end of month 9. Put another way, these factors are candidate moderators of the effect of the implementation strategies. We had also identified the existence of a health system’s opioid prescribing policy as a candidate variable, but since both systems in the trial have such policies, the study offers no variation. Qualitative methods will be used to address two other important questions: (1) how the implementation strategies that make up systems consultation can be further specified, including adaptations made during the trial, and (2) whether a quantitative assessment can be developed to help decision-makers tailor systems consultation to different contexts. A tailoring assessment will be developed, based on a literature review and the findings of the pilot study [18, 21], to assess whether other system-, clinic-, and prescriber-level contextual factors also ought to be considered as tailoring variables in the decisions concerning whether to offer PF and PPC. We will then test the usefulness of these additional variables in the analysis of the trial.

## Methods

### Trial design

**Fig. 2:**
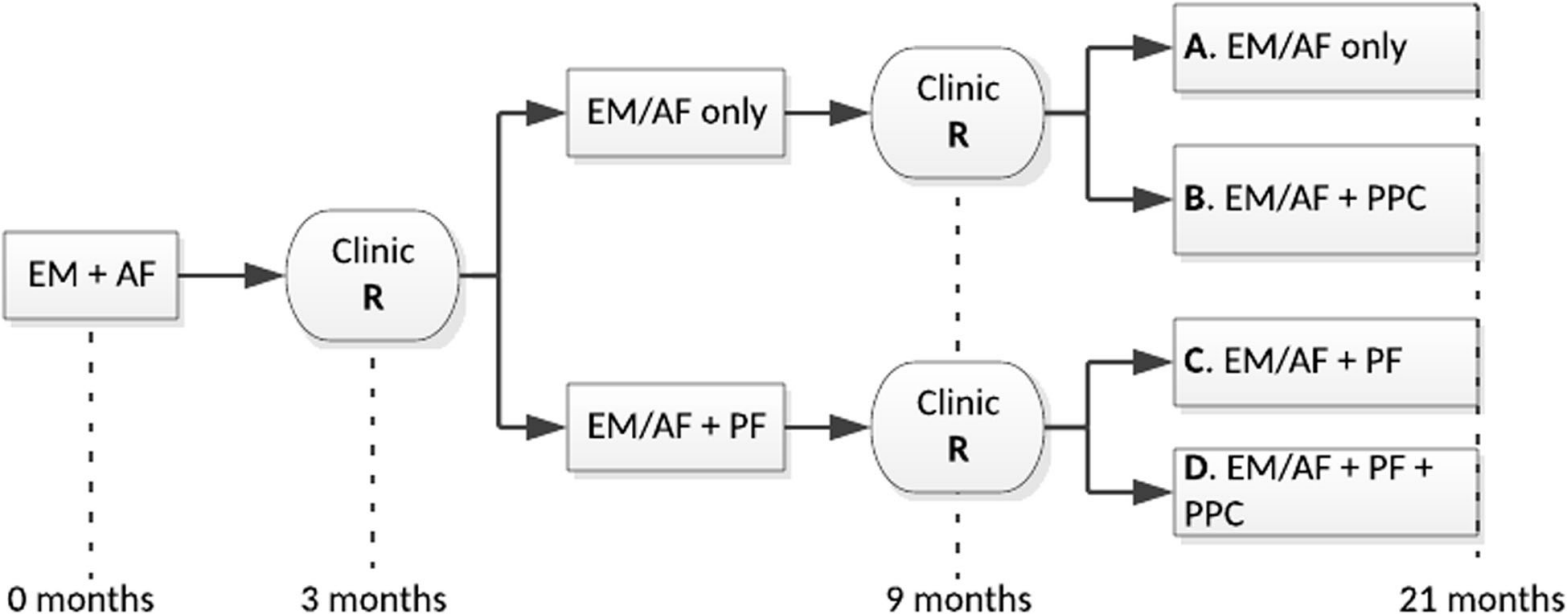
Study design. EM: Educational/engagement meeting; AF: Audit with monthly feedback reports; R: Randomization point; PF: Practice facilitation; PPC: prescriber peer consulting

The trial is an unrestricted, 2 × 2, clustered, sequential, multiple-assignment, randomized trial (SMART) [25–27] (see Fig. 2). Because our goal is to test the effectiveness of the components of an implementation strategy while gathering clinical data related to patient outcomes, the study can be viewed as a hybrid type 3 effectiveness-implementation trial design [30]. All clinics will receive EM/AF to start. At the end of 3 months, half of clinics will be randomly assigned to receive PF for 18 months. At the end of 9 months, a second randomization occurs where half of clinics will receive PPC for 12 months in addition to previously assigned strategies. Clinics will have equal probability of being assigned to one of the four implementation sequences represented by the boxes (A, B, C, or D) on the right side of Fig. 2.

### Setting

We are recruiting primary care clinics from two health systems in the Midwestern USA. One health system operates in the northeast region of Wisconsin and the southern part of the Upper Peninsula of Michigan, which are predominantly rural areas that have been particularly hard hit by prescription opioids. The second health system operates in the Madison, Wisconsin, metropolitan area and the surrounding, predominantly rural region of southcentral Wisconsin. Efforts to interest and involve clinics in the trial took place in the fall of 2019, and a run-in period started with the first educational/engagement meeting on February 13, 2020. The run-in period is planned to end and recruitment to close on May 13, 2020, when randomization takes place.

#### Clinic inclusion/exclusion criteria

**Fig. 3:**
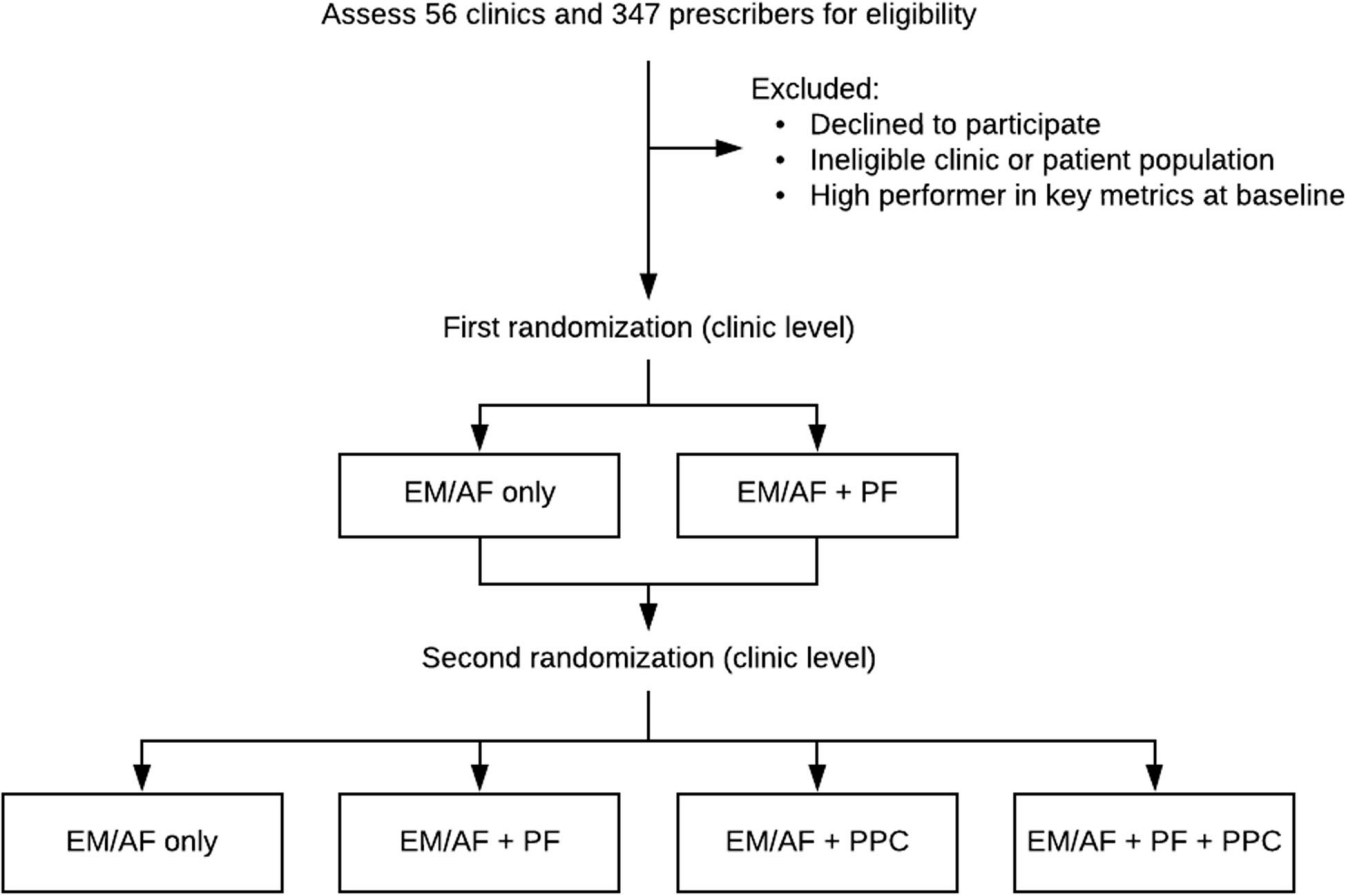
Participant flow. EM, Educational/engagement meeting; AF, Audit with monthly feedback reports; PF, Practice facilitation; PPC, prescriber peer consulting

Only primary care clinics (non-pediatric primary care, internal medicine, and family medicine) will be approached to participate. Clinics that explicitly prohibit initiating opioid therapy will be excluded (e.g., some clinics require that opioids be initiated by a specialty pain clinic). At baseline (before the start of EM/AF), a clinic will be considered ineligible if it already shows exemplary performance on key measures of guideline concordance and would thus receive no benefit from the implementation support we would provide. Specifically, we define a clinic as ineligible if it meets these criteria: (1) 80% or more of a clinic’s long-term opioid patients have treatment agreements and a urine drug screen in the last 12 months and (2) fewer than 10% of the clinic’s patients on long-term opioid therapy have doses above 90 MME. Figure 3 shows the participant flow.

#### Prescriber inclusion/exclusion criteria

Prescribers must be primary care physicians or other providers with prescribing privileges (e.g., nurse practitioners, physician assistants). We will exclude “float” providers (temporary physicians who do not manage stable panels of patients).

#### Patient inclusion/exclusion criteria

Patients included in the calculation of the prescriber-level outcome will have three consecutive months with an opioid prescription in the most recent 3 months documented in the electronic health record, indicating long-term opioid use. We will exclude patients from the calculation who have a cancer diagnosis or are receiving hospice care.

### Randomization and stratification

After 3 months of EM/AF, eligible clinics will be stratified by health system, average number of patients at the clinic prescribed opioids over the first 3 months (above or below the median), and average MME over the first 3 months (above or below the median); clinics will then be randomly assigned with equal probability to PF or no PF arms within each of the eight stratum. At 9 months, clinics will again be stratified by health system, by average number of patients at the clinic prescribed opioids over the past 3 months (median cut) and by average MME over the past 3 months; clinics within each of the resulting strata will be randomly assigned with equal probability to PPC or no PPC arms. The project statistician will generate the random allocation sequence using a random number generator to perform block randomization with blocks of two and four. The study coordinator will enroll clinics and assign them to their randomized group. Consents will be obtained from prescribers and other clinic staff who participate in study activities.

### Implementation strategies

#### Educational/engagement meetings

Educational/engagement meetings (EM) are conceived as a low-intensity, *system-level* implementation strategy. EM involves a broadcast model of communication, involving one or two experts imparting information to many clinicians at once. Educational/engagement meetings will take place at the beginning of the study and then quarterly; a total of six will take place during the 21-month intervention period. The first will be a regionally hosted, in-person training session for each health system; attendees will have the option of participating via webinar. Before the first educational/engagement meeting, the implementation team will ask each clinic’s medical director to identify a change team leader to work with a group of three to seven clinic staff members on improving workflows related to opioid prescribing, and ask the medical director and change team leader to identify other members of the clinic’s change team. We will ask each clinic’s medical director, clinic manager, and change team leader to attend the educational/engagement meeting, minimally, but all change team members and others involved in clinic workflows related to opioid prescribing—prescribers (physicians, nurse practitioners, physician assistants), nurses, medical assistants, lab techs, and so on—will be invited. The educational/engagement meeting will be led by physicians from the University of Wisconsin-Madison with expertise in primary care and addiction medicine and extensive experience managing the care of long-term opioid patients. The session will be designed both to impart information and elicit early engagement and enthusiasm from clinic staff.

As part of educational/engagement meetings, the implementation team will ask system leaders and the medical directors of participating clinics about the challenges they face in opioid prescribing for patients with chronic non-cancer pain. Their responses will be used to focus each presentation on the issues most salient to each system. In addition to covering the current status of opioid prescribing in the system, the sessions will explain how implementation efforts address key issues identified by participants and include time for questions and answers. Subsequent educational/engagement meetings will take place as webinars, be led by physician experts, and cover such topics as effective tapering, opioid rotation, and balancing goals for quality of life, pain, and opioid dose.

#### Audit and feedback

The AF implementation strategy involves system-generated performance feedback reports being sent to participating clinicians. System- and clinic-level feedback reports will be introduced at the first educational/engagement meeting to initiate AF and referred to in all subsequent meetings. After the first meeting, a data coordinator at each clinic (a change team member asked to assume this role) will access and distribute monthly feedback reports to other change team members, prescribers, and the clinic’s medical director and manager.

The combination of EM and AF represents a blended, system-level implementation strategy. While system-level implementation strategies are relatively inexpensive and easy to scale, such strategies have limited evidence of effectiveness [17]. Nonetheless, simply learning and being convinced about what to do with respect to opioid prescribing—and having access to performance data that can be used to guide changes—may suffice to improve prescribing in some clinics. Moreover, the provision of subsequent strategies (e.g., PF and PPC) is expected to build synergistically on the foundational knowledge and reports generated by EM and AF, respectively.

#### Practice facilitation

Practice facilitation (PF) is a *clinic*-*level* strategy that targets clinic processes and workflows. In general, practice facilitation focuses on local customization and has a stronger evidence base than educational/engagement meetings and audit and feedback reports, which are usually not tailored to specific clinics [31]. Practice facilitation is also more labor intensive than educational/engagement meetings and audit and feedback reports. Solving workflow problems (e.g., streamlining processes around opioid prescription refills), which practice facilitation addresses, may be the key to improvement in some clinics.

In a clinic randomized to receive practice facilitation, an external change agent trained in practice facilitation (the facilitator) will visit the clinic in-person, and then follow-up over the course of five monthly and four quarterly videoconferences or teleconferences to help clinics improve processes related to opioid prescribing, such as (1) ensuring that treatment agreements are initiated and regularly updated and (2) integrating urine drug testing into clinic workflows. Clinic change teams consisting of a change team leader, a data coordinator responsible for distributing AF reports, at least one prescriber, and up to four other staff members will form the change team for opioid prescribing. The facilitator will work with the change team to use systems engineering tools (e.g., walkthrough exercises, flowcharting, and nominal group technique [32, 33]) to make changes in clinic workflows. The facilitator will reinforce the content of the EMs and guide teams in using their clinic and prescriber level AF reports to monitor progress towards goals.

#### Prescriber peer consulting

Prescriber peer consulting (PPC) is a *prescriber*-*level* strategy that aims to help prescribers manage their patients on long-term opioids by providing the opportunity to consult with a physician experienced in opioid management. PPC will be available to all prescribers at clinics randomized to receive this strategy; hence, it is conceptualized in this trial as a clinic-level strategy in terms of its delivery. Peer consultants will be physicians or pharmacists with relevant experience in opioid prescribing and addiction medicine nominated by health system leaders to help their peers manage patients on long-term opioid therapy (e.g., how to manage the tapering of opioid doses for long-term opioid patients with clinically indicated dose reductions). Participating prescribers in clinics randomized to PPC (including nurse practitioners and physician assistants) will receive up to four quarterly consulting sessions over 12 months. Consultations will be delivered via videoconference or teleconference. Prescribers may choose to include other staff at their clinic (e.g., RNs, MAs) in these consultations as well. Prescriber peer consulting is highly resource intensive, but our preliminary research suggests that physician-to-prescriber interaction may be the most effective way to change prescribing behavior.

### Measures and outcomes

The study uses the RE-AIM model as an evaluation framework [34]. RE-AIM is a comprehensive evaluation framework that assesses five dimensions: Reach, Effectiveness, Adoption, Implementation, and Maintenance. Specific measures for each RE-AIM dimension are presented in Table 1. Evaluation data will come primarily from electronic health records (EHRs). Both health systems use Epic Systems’ EHR, which will facilitate the extraction of EHR data. One system was the site of our pilot research. Detailed specifications were developed during the pilot that will be used to ensure consistent data definitions across both systems. The primary outcome is prescriber average of MME dose per day per opioid patient, calculated over a 3-month period.

**Table 1:** Outcome measures by RE-AIM category

Domain	Source	Months* collected
**Reach:** # and % of patients excluded vs. participating (incl. characteristics)	EHR	1–21
**Effectiveness:** prescriber averages of MME dose per day per opioid patient over the past 3 months (primary outcome)	EHR	1–21
# and % of patients completing urine drug testing (past 12 months)	EHR	1–21
# and % of patients screened for mental health using PHQ-2 (past 12 months)	EHR	1–21
Mental health (PHQ-9) scores for patients screening positive on PHQ-2 (past 12 months)	EHR	1–21
Overall rate and dose of opioid-benzodiazepine co-prescribing	EHR	1–21
# and % of patients with treatment agreements (past 12 months)	EHR	1–21
# and % of opioid prescriptions above 90 MME	EHR	1–21
Patient attendance at scheduled clinic visits	EHR	1–21
# and % of patients prescribed buprenorphine	EHR	1–21
# and % of patients with PEG-3 score (past 12 months)	EHR	1–21
PEG-3 scores (past 12 months)	EHR	1–21
**Adoption (setting):** # and % of participating clinics vs. all clinics (incl. characteristics)	Health system	1–21
**Adoption (staff):** # and % of participating staff vs. all eligible clinic staff (incl. characteristics)	Clinic	1–21
Clinician attendance at intervention meetings	Research team	1–21
**Implementation:** Hours of intervention received per clinic and prescriber	Research team	1–21
Adaptations made to protocols during intervention period	Research team	1–21
Assessment of moderators: clinic-level experience in QI, size of clinic (# patients), # and % of patients at the clinic on opioid doses > 90 MME	Research team, EHR	0, 3, 9, 21
Qualitative assessment of mechanisms of action and factors influencing implementation	Research team	1–21
Cost of each implementation sequence and combination	Research team	1–21
**Maintenance:** 6-month follow-up on all effectiveness outcomes	EHR	22–27

*EHR* electronic health record

*Months correspond to intervention months

### Primary aim analyses

All clinics randomized at the end of month 3 will be included in the intent-to-treat sample for all aims. The primary research outcome, MME, will be available for all prescribers within all clinics that consent to be in the study (approximately 6 per clinic). Table 2 shows the sequences of implementation strategies that will be employed in the trial.

**Table 2:** Sequences of implementation strategies

Conditions (Fig. 2)	Sequence of implementation strategies (*A*_1_*, A*_2_)	Intervention months 0–3	Intervention months 4–9	Intervention months 10–21
A	EM/AF only (− 1, − 1)	EM/AF	No PF	EM/AF
B	EM/AF + PPC (− 1,+ 1)	EM/AF	No PF	EM/AF + PPC
C	EM/AF + PF (+ 1,− 1)	EM/AF	Add PF	EM/AF + PF
D	EM/AF + PF + PPC (+ 1,+ 1)	EM/AF	Add PF	EM/AF + PF + PPC

For the primary aim, we will determine the effect of strategy sequence D (the most intensive sequence of strategies) vs. strategy sequence A (the least intensive strategy) on change in MME from intervention months 3 to 21. Strategy sequence D offers EM/AF during months 3–21, augments with PF during months 4–21, and then further augments with PPC during months 10–21. By contrast, strategy sequence A offers EM/AF but never offers PF or PPC. This analysis is a mean comparison of change in MME between strategy sequences D versus A. The analysis will use a longitudinal (repeated-measures) analysis. Time will be coded such that *t =* 0 denotes the end of month 3 of the intervention period (the initial randomization); in the following text, data collected prior to *t =* 0 is considered baseline data. The primary outcome (MME) is a continuous measure and is collected at intervention month 3 (*t* = 0, immediately prior to randomization) and every month up to intervention month 21 (*t* = 18). The primary outcome is an average over 3 months; thus, there are a total of 7 measurement occasions. This is a 3-level analysis: repeated measures of MME, within prescribers, within clinics.

A piecewise-linear model with a knot at intervention month 9 (*t =* 6, MME collected immediately before the second randomization) will be used to model the temporal trajectories over the course of intervention months 4–21. Equation 1 displays the planned longitudinal model we will use to model the mean MME over time and test the primary aim.

Equation 1 Longitudinal model for mean MME

1 $$ {\displaystyle \begin{array}{l}{\eta}^{\prime }X+{\gamma}_0+{I}_{\left(t\le 6\right)}\left({\gamma}_1t+{\gamma}_2{tA}_1\right)\\ {}\kern4.199998em +{I}_{\left(t>6\right)}\left(6{\gamma}_1+6{\gamma}_2{A}_1+{\gamma}_3\left(t-6\right)+{\gamma}_4\left(t-6\right){A}_1+{\gamma}_5\left(t-6\right){A}_2+{\gamma}_6\left(t-6\right){A}_1{A}_2\right)\end{array}} $$

*X* is the mean-centered baseline covariates (clinic aggregate MME at *t* = 0 and a dummy indicator for health system), *A*_1_ is the indicator for the first randomization (PF = 1 vs. no PF = − 1), and *A*_2_ denotes the second randomization (PPC = 1 vs. no PPC = − 1). The model has a linear trend from *t* = 0 to *t* = 6 for prescribers in PF and no PF clinics, and a linear trend from *t* = 6 to month *t* = 18 for each of the four sequences of strategies (A–D). We allow for changes in the mean trajectory (i.e., deflections) at intervention month 9 (*t* = 6) since this is the point at which clinician prescribers may begin receiving PPC. *γ*_0_ is the mean outcome at intervention month 3 (*t* = 0), averaged across all four strategy sequences; *γ*_1_ is the average change in MME from month 3 (*t* = 0) to month 9 (*t* = 6), averaged across all four strategy sequences; 2*γ_2_ is the causal effect of PF vs. no PF on change in MME from intervention month 3 (*t* = 0) to intervention month 9 (*t* = 6); *γ*_3_ represents the average change in MME from intervention month 9 (*t* = 6) to intervention month 21 (*t* = 18), averaged across all four strategy sequences; 2*γ_4_ is the main causal effect of PF vs. no PF on change in MME from month 9 (*t* = 6) to month 21 (*t* = 18), averaged over PPC vs. no PPC; 2*γ_5_ is the main causal effect of PPC vs. no PPC on change in MME from month 9 (*t* = 6) to month 21 (*t* = 18), averaged over PF vs. no PF; *γ*_6_ is the interaction term to quantify whether and how PF and PPC work together to impact change in MME from month 9 (*t* = 6) to month 21 (*t* = 18).

The planned statistical test associated with the primary aim (for which we power the study) is a test of the null hypothesis that 12*γ*_2_ + 24*γ*_4_ + 24*γ*_5_ = 0, that is, that there is no difference on change in MME from month 3 (*t* = 0) to month 21 (*t* = 18) between implementation sequence A vs. implementation sequence D. We will report estimates of each coefficient in the model with their corresponding 95% confidence intervals.

Since the covariates *X* and the primary outcome data are available and passively collected from the EHR, except in rare cases (e.g., clinician turnover, clinician retirement, or an error leading to data loss in the EHR), we expect to have little missing data.

Additional file 1 describes the planned analysis for the primary outcome in more detail.

#### Sample size and power

The total sample size for this study is based on the primary aim: a comparison on average difference on change in MME from intervention month 3 (*t* = 0) to intervention month 21 (*t* = 18) between implementation sequence D vs. implementation sequence A. This is a comparison between two of the four groups embedded in the trial (see Table 2). The sample size calculator for this comparison is a straightforward adjustment to the sample size calculator for a standard two-sample hypothesis test. The adjustment accounts for the clustering of prescribers within clinics through a variance inflation factor (VIF) of 1 + (*m* − 1) *ρ*, where *m* is the (average) number of prescribers per clinic and *ρ* is inter-clinic correlation coefficient (ICC) for MME at month 21 (*t* = 18). Based on intervention clinics in the R34 pilot data, the ICC was estimated to be *ρ* = 0.14. Assuming an average of *m* = 6 prescribers per clinic (based on information from the new health systems that have agreed to participate), a Type-1 error rate of *α* = 5% and *ρ* = 0.14, a minimum of 64 prescribers in each group (11 clinics per group) will provide at least 80% power to detect a moderate effect size of *d* = 2/3 between the two implementation sequences on change in MME. Because we have four groups in this trial, the minimum total study sample size is 256 clinician prescribers, corresponding to roughly *N* = 40–45 clinics (depending on prescriber count).

Based on the pilot data that found a standard deviation of 35 for MME, an effect size of *d* = 2/3 corresponds to detecting an average difference of at least 23 on the MME between the two implementation sequences after 21 months. The above calculation is expected to be conservative because it does not account for within-prescriber correlation in MME, which is accounted for in the longitudinal analyses and could permit detection of smaller differences in MME.

### Exploratory aim

Q-learning [35]—a generalization of moderated regression analysis to multiple stages of implementation—will be used to test the moderators and construct a candidate adaptive implementation strategy.

### Secondary aim

In the secondary aim, we will estimate the cost of delivering four different sequences and combinations of strategies (EM/AF, EM/AF + PF, EM/AF + PPC, and EM/AF + PF + PPC), including the incremental cost-effectiveness of adding facilitation and prescriber peer consulting. Results will help decision-makers weigh the costs and effects of using different sequences of implementation strategies.

In line with the pragmatism that underlies this research, we will employ an operational cost analysis based on tenets of engineering economics. Traditional health economic approaches incorporate concepts from welfare economics and take a societal perspective towards decision analysis [36, 37]. Engineering economic analysis tends to have a narrower scope. Whereas health economic evaluation provides information primarily for policymakers, engineering economic analysis produces information primarily for the organizational leaders who ultimately make decisions about the adoption of evidence-based practices in their organizations. We adopt the perspective of the healthcare system (rather than society at large) in considering the incremental costs and effects associated with ratcheting up the implementation strategy. This perspective deemphasizes some societal costs (e.g., patient travel time) and effects (e.g., crime related to addiction) that are often considered in traditional cost-effectiveness analysis [38]. However, the health system perspective aligns with updated guidelines for cost-effectiveness that were re-issued in 2016 [39] that acknowledge the importance of the health care perspective for pragmatic purposes. The health systems we will work with, like many health systems, are Accountable Care Organizations, which means that they are responsible for their patients’ total cost of care. The pragmatic optimization approach featured in this aim was designed in close partnership with our research collaborators to model the considerations healthcare decision-makers told us they actually use when making decisions about adopting and sustaining evidence-based practices.

We developed an approach to costing the systems consultation strategy in our pilot research [18]. Detailed logs were kept of all contacts between members of the research team and the clinic change teams to estimate the number of hours spent delivering systems consultation. These estimates were multiplied by hourly wage rates for physician consultants and the facilitators. Costs for clinic incentives (continuing education credits, clinic stipends) and expenses associated with site visits were also included in the cost assessment. The total cost of delivering the entire systems consultation implementation strategy for 6 months (i.e., the full package of strategies corresponding to box D in Fig. 2) was estimated in the pilot research. The log-based costing approach is sufficiently fine-grained to construct detailed breakdowns of unit costs associated with each component (EM/AF, PF, and PPC) of the full systems consultation intervention [18].

We will use incremental cost-effectiveness ratios (ICERs) to quantify the tradeoff between the additional effectiveness achieved through scaling up the intensity of implementation strategies. The primary ICER will be the incremental cost per unit reduction in MME. We will use a 21-month timeline for the cost analysis. Implementation costs will be organized using the Cost of Implementing New Strategies framework [38] and categorized under the “Implementation” domain of RE-AIM (see Table 1). Secondary ICERs will include incremental cost per unit change in prevalence of opioid/benzodiazepine co-prescribing, completion of treatment agreements, urine drug screens, and so on, as shown in Table 1. Cost-effectiveness acceptability curves will be generated (using Monte Carlo simulation techniques) for all primary and secondary ICERs to model uncertainty in our estimates of cost-effectiveness [39].

### Trial status

Sites were identified and participation confirmed by January 31, 2020. Site training began with the first educational/engagement meetings, which were held in February and March 2020. The trial was temporarily suspended on March 25, 2020, because the coronavirus pandemic took priority in both health systems.

## Discussion

Although much is known about *what* to do at the patient level to more safely prescribe and monitor opioids for chronic, non-cancer pain, *how* to implement these practices among prescribers remains a challenge. The literature offers virtually no guidance on which implementation strategies are most effective in different clinical contexts. This study collects data that can be used to empirically develop a multi-level, adaptive, scalable implementation strategy called *systems consultation*, which is a blend of discrete implementation strategies. The goal of the study is to understand the optimal sequencing and combination of implementation strategies that specific types of clinics and prescribers need to adopt clinical guidelines for opioid prescribing. Specifically, we will test four different sequences and combinations of strategies (EM/AF, EM/AF + PF, EM/AF + PPC, and EM/AF + PF + PPC) to assess the effectiveness and cost of augmenting a systems-level, broadly based implementation strategy (EM/AF) with progressively more intensive strategies.

The proposed study uses a novel clustered SMART design, which allows us to compare the different sequences of strategies over time in real-world primary care clinics. The design allows us to document the value added to the least intensive strategy by more intensive strategies. The SMART also enables us to construct empirically an adaptive implementation strategy to meet the needs of different clinics. On the PRECIS-2 continuum of trials from explanatory to pragmatic, the study falls at the pragmatic end [40].

Despite the strengths of the study, it also has limitations. All eligible clinics in two health systems were invited to participate. The clinics that volunteered may be more motivated to change than those that do not, limiting the generalizability of the findings. The two health systems in which the study will be conducted serve relatively rural regions with less racial and ethnic diversity than are common in other parts of the USA. Study data will come from data in the electronic health record. Pragmatic data collection of this kind eases the burden of data collection on research participants, but may offer comparatively limited understanding compared with using data from validated instruments.

## Conclusions

The design of the current study will enable us to learn how to tailor implementation strategies so that, ideally, clinics and prescribers can receive exactly the implementation support they need, when they need it. The pragmatic trial we propose may be generalized to other regions of the USA struggling with prescription opioids, and potentially to other significant public health issues. Results of the study, when combined with results from other trials also focusing on adaptive implementation strategies, will increase our knowledge of how to tailor implementation strategies to different settings, ultimately increasing the speed and efficiency of delivering evidence-based practices into health systems.

## Supplementary information

**Additional file 1:.** Supplementary appendix: Balanced Opioid Initiative trial design and primary analysis protocol

**Additional file 2:.** CONSORT checklist for cluster-randomized trials

**Additional file 3:.** StaRI Checklist

**Additional file 4:.** Proof of ethics

**Additional file 5:.** Proof of funding

## Data Availability

The datasets used and/or analyzed during the current study will be available from the corresponding author on reasonable request.

## Abbreviations

AF: Audit and feedback
EBP: Evidence-based practice
EM: Educational/engagement meeting
EHR: Electronic health record
MME: Morphine-milligram equivalent dose
PF: Practice facilitation
PPC: Prescriber peer consulting
RE-AIM: Reach, Effectiveness, Adoption, Implementation, and Maintenance
SMART: Sequential, multiple-assignment randomized trial
