# Supplementary material for: The Balanced Opioid Initiative: protocol for a clustered, sequential, multiple-assignment randomized trial to construct an adaptive implementation strategy to improve guideline-concordant opioid prescribing in primary care
Source: Implement Sci. 2020 Apr 25;15:26. doi: 10.1186/s13012-020-00990-4 (PMC7183389; doi:10.1186/s13012-020-00990-4)
Supplement: Supplementary file 1 — Additional file 1:. Supplementary appendix: Balanced Opioid Initiative trial design and primary analysis protocol [file 13012_2020_990_MOESM1_ESM.pdf]

Supplementary Appendix: Balanced Opioid Initiative Trial Design and Primary Aim Analysis Protocol

Daniel Almirall   Nicholas Schumacher   Andrew Quanbeck   James Robinson

March 27, 2020

This supplementary appendix describes additional details concerning the data collection protocol, the definition of the study's primary outcome, how the randomizations are stratified, the study's primary aim analysis, and total sample size calculation for the study. The details contained herein are not essential for the reader to understand the protocol manuscript; rather, they are designed for readers who want additional detail concerning the data collection scheme and primary aim analysis. The contents herein also serve as a complete pre-specification (prior to the completion of data collection) of the data collection and the primary aim data analysis. Note the specific focus of this supplement is on the study's primary aim; we do not provide detail for the secondary aim analyses.

1 Source of Data, Notation and Set-up

Data is to be extracted monthly from the electronic health record at each of the two health systems. A total of 30 data extractions will occur, at time points $t = -2, -1, 0, 1, 2, \ldots, 27$. The data extraction at each time t will pull clinic, provider, patient data related to opioid use and related factors over past month (since last data pull).

- There are five phases to the 30-month study. A 3 month pre-implementation phase, 3 stages of implementation totaling 21 months, and a follow-up 6-month period:
  1. Pre-implementation: months ending at $t = -2, -1, 0$
  2. Stage 1 implementation: months ending at $t = 1, 2, 3$
  3. Stage 2 implementation: months ending at $t = 4, \ldots, 9$
  4. Stage 3 implementation: months ending at $t = 10, 11, \ldots, 21$
  5. Follow-up period: months ending at $t = 22, \ldots, 27$.
- j denotes clinic, $j = 1, 2, \ldots, J$
  - All J clinics that meet inclusion criteria are known at $t = 0$. To be included in the intent-to-treat sample, clinic j must meet the following criteria: 1) at least one clinician from clinic j must attend the educational meeting at $t = 0$; or 2) clinic j communicates their change team member's names to the study team and at least one clinician from clinic j watches a recording of the educational meeting presented at $t = 0$.
  - We expect to recruit $J = 44$ clinics in total across two health systems, see Section 6 below.
  - A unique clinic identification number will follow the J clinics throughout all data extractions
- i denotes clinician prescriber, $i = 1, 2, \ldots, N_j$ within clinic j
  - There are N_j clinicians who meet inclusion criteria per clinic j at $t = -2$.

- * Note that N_j at each clinic j does not vary with time. The total sample of clinician prescribers N is known before the trial's first randomization, at $t = -2$; or earlier. This is consistent with the primary aim analysis as an assessment of the 4 implementation strategies on the marginal mean of a prescriber level outcome.
- Based on prior data, the average number of clinician prescribers per clinic is $N_j \approx 6$
- We expect there to be at least $N = 256$ clinician prescribers in total, see Section 6
- These $N = \sum_j^J N_j$ clinicians are all known at $t = -2$
- A unique clinician (within clinic) identification number will follow the N prescribers throughout all 30 data extractions
- Clinicians leaving a clinic: Clinicians are identified throughout the study with the clinic to which they belong at time $t = -2$, even if they switch from one clinic to another within the same health system; this is expected to occur rarely, if at all. Even if a clinician leaves the clinic for another one within the same health system, we have a plan for obtaining their research outcomes related to opioid prescribing.
- Clinicians entering a clinic: We will collect data on new clinicians that enter a clinic after $t > -2$, but they are not part of the primary aim analysis; this is expected to occur rarely, if at all.

- P_{jit} denotes the total number of unique patients who meet study inclusion criteria and who clinician i prescribed an opioid to in clinic j at any time in the month leading to time t
  - Note this is the total number of unique patients, not total patient encounters which may be larger (e.g., if at least one of the patients received a prescription twice in one month)
  - Note that $P_{j,i,t}$ may be zero (this is expected to happen rarely in the target population of clinician prescribers N) but it cannot be negative (as it is a count).
- $\mathbb{1}_{(P_{jit}>0)}$ is a binary indicator which equals 1 if $P_{jit} > 0$ or 0 if $P_{jit} = 0$.
  - $P_{jit} = 0$ should not be conflated with the idea that clinician data is missing (e.g., clinician has dropped out of the clinic). Rather, $P_{jit} = 0$ means that clinician i in clinic j did not prescribe to any new patients in the month leading to time t. Missingness would, instead, mean that we do not have the value of P_{jit} available. See Section 5 for a discussion of missing data.
- M_{jit} denotes the total sum of morphine milligram-equivalent per day (MME) that the clinician i in clinic j prescribed to all P_{jit} patients in the month leading to time t
  - Note that M_{jit} is already calculated as the *per day* MME
- $H_j = H_{jit}$ denotes health system type for clinic j; this variable does not vary by time or clinician within clinic; it is known at $t = -2$ and does not change

2 Primary Outcome

The primary outcome for clinician i in clinic j at time t, denoted Y_{jit} (at $t = 3, 6, \ldots, 21$) is defined as

$$Y_{jit} = \frac{1}{\left(\sum_{k=0}^{2} \mathbb{1}_{(P_{j,i,t-k}>0)}\right)} \left(\mathbb{1}_{(P_{j,i,t-2}>0)} \frac{M_{j,i,t-2}}{P_{j,i,t-2}} + \mathbb{1}_{(P_{j,i,t-1}>0)} \frac{M_{j,i,t-1}}{P_{j,i,t-1}} + \mathbb{1}_{(P_{j,i,t}>0)} \frac{M_{jit}}{P_{jit}} \right).$$

Each Y_{jit} ($t = 3, \ldots, 21$) is a measure of the average MME per day prescribed by the clinician over the course of 3 months. Note that the $P_{j,i,t}$'s in the denominator may differ from month to month over the 3-month period; and, as discussed above, it may be equal to zero. Note that the indicator $\mathbb{1}_{(P_{jit'}>0)}$ is included

so that if a clinician does not write any prescriptions in the interval leading to time t' (i.e., $P_{jit'} = 0$) then the contribution to the sum for that time point is zero (i.e., $\mathbb{1}_{(P_{jit'}>0)} \frac{M_{j,i,t'}}{P_{jit'}} = 0$). In most cases, $\left(\sum_{k=0}^{2} \mathbb{1}_{(P_{j,i,t-k}>0)}\right)$ will be equal to 3.

3 Stratified Randomizations

The first randomization to PF vs no PF at $t = 3$ (e.g., May 1, 2020) is to be stratified based on three variables

- $H_j = \mathbb{1}_{\text{Bellin}}$: a binary indicator of health system type which equals 1 if Bellin Health System or 0 if UW Health System (no other health systems are participating).
- $\tilde{P}_{j,1,2,3} = \frac{1}{3}\sum_i^{N_j}(P_{ji1} + P_{ji2} + P_{ji3})$: the total number of patients prescribed opioids at clinic j, averaged over the past 3 months.
- $\tilde{Y}_{j,1,2,3} = \frac{1}{\left(\sum_{k=0}^{2} \mathbb{1}_{(P_{j,i,t-k}>0)}\right)N_j} \sum_i^{N_j}\left(\mathbb{1}_{(P_{ji1}>0)}\frac{M_{i,j,1}}{P_{ji1}} + \mathbb{1}_{(P_{ji2}>0)}\frac{M_{ji2}}{P_{ji2}} + \mathbb{1}_{(P_{ji3}>0)}\frac{M_{ji3}}{P_{ji3}}\right)$: the average MME per day over the past 3 months for clinic j.

For $\tilde{P}_{j,1,2,3}$ and $\tilde{Y}_{j,1,2,3}$, we plan to set the cutoff at the health system specific median. This will lead to $2^3 = 8$ strata. Randomization lists will be created within each of these 8 strata using blocks of size 2 and 4 (in random order). Only the study coordinator will have access to the randomizations lists.

Similarly, the second randomization to PPC vs no PPC at $t = 9$ (e.g., November 1, 2020) is to be stratified based on

- $H_j = \mathbb{1}_{\text{Bellin}}$: a binary indicator of health system type, as above
- $\tilde{P}_{j,7,8,9} = \frac{1}{3}\sum_i^{N_j}(P_{ji7} + P_{ji8} + P_{ji9})$: the total number of patients prescribed opioids at clinic j, averaged over the past 3 months.
- $\tilde{Y}_{j,7,8,9} = \frac{1}{\left(\sum_{k=0}^{2} \mathbb{1}_{(P_{j,i,t-k}>0)}\right)N_j} \sum_i^{N_j}\left(\mathbb{1}_{(P_{ji7}>0)}\frac{M_{i,j,7}}{P_{ji7}} + \mathbb{1}_{(P_{ji8}>0)}\frac{M_{ji8}}{P_{ji8}} + \mathbb{1}_{(P_{ji9}>0)}\frac{M_{ji9}}{P_{ji9}}\right)$: the average MME per day over the past 3 months for clinic j.

Again, for $\tilde{P}_{j,7,8,9}$ and $\tilde{Y}_{j,7,8,9}$, we plan to set the cutoff at the health system specific median. This will again lead to $2^3 = 8$ strata. Randomization lists will be created within each of these 8 strata using blocks of size 2 and 4 (in random order). Again, only the study coordinator will have access to the randomizations lists.

4 Primary Aim Analysis

All J clinics that are randomized at $t = 3$ (the first randomization) and all $N = \sum_j^J N_j$ prescribers within these clinics (these prescribers are all known at $t = 0$) will be included in the intent-to-treat sample for the primary aim analysis; this intent-to-treat sample includes clinics (and prescribers within clinics) who do not adhere to implementation, drop-out of the implementation, or drop-out of the study after randomization at $t = 3$.

The study design is an unrestricted, clustered, 2×2 sequentially-randomized trial; sequential randomizations are at the level of the clinics j. This type of sequentially-randomized trial leads to four sequences of implementation. Table 1 shows the four sequences of implementation implementations that are embedded as part of this study, which we denote by the pair $(A_{j,1}, A_{j,2})$, where $A_{j,1}$ denotes whether (1) or not (-1) PF; and $A_{j,2}$ denotes whether (1) or not (-1) PPC.

Table 1: Four sequences of implementation implementations embedded in the study.

**Implementation Intervention Sequence**	**Stage 1** $t = 1, 2, 3$ **Begin With**	**Stage 2** (A_1) $t = 4, \ldots, 9$ **Augment With**	**Stage 3** (A_2) $t = 10, \ldots, 21$ **Augment With**	**Condition (in Figure 1 of manuscript)**
EM/AF ($A_1 = -1, A_2 = -1$)	EM/AF	–	–	A
EM/AF+PPC ($A_1 = -1, A_2 = 1$)	EM/AF	–	PPC	B
EM/AF+PF ($A_1 = 1, A_2 = -1$)	EM/AF	PF	–	C
EM/AF+PF+PPC ($A_1 = 1, A_2 = 1$)	EM/AF	PF	PPC	D

Notes. EM, Educational meeting; AF, Audit and feedback; PF, Practice facilitation; PPC, Prescriber Peer Coaching; – means implementation not augmented

4.1 Primary Aim

The primary aim analysis will use the outcomes Y_{jit} ($t = 3, 6, \ldots, 21$), that is, average 3-month prescriber-level MME from the first 3 months of implementation (the interval during which all clinics were on EM/AF alone) ($t = 1, 2, 3$, pre-randomization data) up to the end of planned implementation ($t = 7, 20, 21$). Y_{jit} is a continuous measure.

The primary aim is a comparison between clinics that are offered the EM/AF+PF+PPC sequence (condition D, the most intensive sequence of implementation strategies) versus EM/AF (condition A, the least intensive strategy) on change in the average Y_{jit} from $t = 3$ to $t = 21$. In the following, we drop the (j, i) notation (but retain the notation for time t) for Y_{jit}, for simplicity. The primary aim targets the following estimand (causal effect):

$$\Delta = E_{(1,1)}\big(Y_{21} - Y_3\big) - E_{(-1,-1)}\big(Y_{21} - Y_3\big) = E_D\big(Y_{21} - Y_3\big) - E_A\big(Y_{21} - Y_3\big) \quad (1)$$

where the $E_{(a_1,a_2)}$ is used to denote the expectation of Y_t had all clinics (and all prescribers within those clinics who are known at $t = -2$) received the implementation implementation sequence $(A_1, A_2) = (a_1, a_2)$. Thus, the primary aim targets the average difference in average 3-month prescriber-level MME from the point of initial randomization to the end of month 21, that is, $E_{(a_1,a_2)}(Y_{21} - Y_3)$, between the groups D ($a_1 = 1, a_2 = 1$) vs. A ($a_1 = -1, a_2 = -1$).

4.2 Model

Let X denote the following two baseline, clinic-level covariates (mean-centered): $(H_j, \tilde{P}_{j,1,2,3})$; that is, health system type (UW Health vs Bellin) and the average number of patients prescribed opioids over the first 3 months, respectively. Since X is mean-centered, then $E(X) = 0$; this will facilitate the interpretation of parameters in the model used below. Next, to further facilitate the interpretation of model parameters, we let $t^* = t - 3$ and we will use $t^* = 0, 3, \ldots, 18$ in the repeated measures data analysis. That is, for the primary aim data analysis we re-define time such that $t^* = 0$ denotes the point of first randomization. Note that the second randomization occurs at $t^* = 6$ (or $t = 9$).

We will use the following piecewise-linear, repeated measures model for $E(Y_{j,i,t^*} \mid X_j, A_{1,j}, A_{2,j})$ as the primary aim analysis model:

$$\mu_{j,i,t^*}(X_j, A_{1,j}, A_{2,j}; \theta) = \eta' X_j + \gamma_0 + I_{(t^* \le 6)}\Big(\gamma_1 t^* + \gamma_2 t^* A_{1,j}\Big)$$
$$+ I_{(t^*>6)}\Big(6\gamma_1 + 6\gamma_2 A_{1,j} + \gamma_3(t^* - 6) + \gamma_4(t^* - 6)A_{1,j} + \gamma_5(t^* - 6)A_{2,j} + \gamma_6(t^* - 6)A_{1,j}A_{2,j}\Big). \quad (2)$$

Note that the marginal mean under this model depends on j only through $(X_j, A_{1,j}, A_{2,j})$. This marginal mean model has nine unknown parameters $\theta = (\eta, \gamma) = (\eta_1, \eta_2, \gamma_0, \gamma_1, \ldots, \gamma_6)$. (η_1, η_2) represent the association between $(H_j, \tilde{P}_{j,1,2,3})$ and MME. The γ parameters can be interpreted as follows:

- γ_0 is the average MME at the point of first randomization, i.e., $t^* = 0$ $(t = 3)$. This is an average across all four implementation implementation sequences.
- γ_1 is the average change in MME during stage 2 implementation, i.e., from $t^* = 0$ to $t^* = 6$ (from $t = 3$ to $t = 9$). This is the average change across all four implementation implementation sequences.
- $2\gamma_2$ is the average causal effect of augmenting implementation with PF vs. not augmenting with PF on change in MME during stage 2 implementation, i.e., from $t^* = 0$ to $t^* = 6$ (from $t = 3$ to $t = 9$).
- γ_3 is the average change in MME during stage 3 implementation, i.e., from $t^* = 3$ to $t^* = 18$ (from $t = 9$ to $t = 21$). This is the average change across all four implementation implementation sequences.
- $2\gamma_4$ is the average causal effect of augmenting implementation with PF vs. not augmenting with PF on change in MME during stage 3 implementation, i.e., from $t^* = 6$ to $t^* = 18$ (from $t = 9$ to $t = 21$). This is a main effect, that is, it is averaged over A_3 (PPC).
- $2\gamma_5$ is the average causal effect of augmenting implementation with PPC vs. not augmenting with PPC on change in MME during stage 3 implementation, i.e., from $t^* = 6$ to $t^* = 18$ (from $t = 9$ to $t = 21$). This is a main effect, that is, it is averaged over A_2 (PF).
- γ_6 is the interaction term used to quantify whether/how PF and PPC work together to cause effect on change in MME during stage 3 implementation, i.e., from $t^* = 6$ to $t^* = 18$ (from $t = 9$ to $t = 21$).

4.3 Estimation

We will employ a three-level (repeated measures for each of the prescribers within each of the clinics) generalized estimating equations regression to estimate the 9 unknown parameters in (η, γ). Specifically, let $Y_j = ((Y_{j,1,0}, \ldots, Y_{j,1,18}), \ldots, (Y_{j,N_j,0}, \ldots, Y_{j,N_j,18}))$ be the "stacked" vector of outcomes for all 7 time points for each of the N_j prescribers within clinic j. Y_j is of size $(7 * N_j) \times 1$. Let $\mu_{j,\theta} = \mu_{j,\theta}(X_j, A_{1,j}, A_{2,j})$ be the appropriately "stacked" marginal mean model vector for Y_j shown in Display (2), also of size $(7*N_j)\times 1$. Let $D_j = D_j(X_j, A_{1,j}, A_{2,j}) = \partial\mu_{j,\theta}/\partial\theta$ be the marginal mean model's derivative with respect to the unknown θ; D_j is a matrix of size $(7 * N_j) \times 9$. We will use an exchangeable working covariance matrix of size $(7 * N_j) \times (7 * N_j)$ for Y_j, defined as $V_{j,(\sigma_Y,\rho,r)} = \sigma_Y^2 R_{j,(\rho,r)}$, where $R_{j,(\rho,r)}$ (working correlation matrix) is given by

$$R_{j,(\rho,r)} = \rho 1_{7*N_j} 1'_{7*N_j} + (r - \rho)\ \mathrm{bdiag}_{N_j}(1_7 1'_7) + (1 - r)\ I_{7*N_j}$$

where 1_{7*N_j} is a vector of 1's of size $(7*N_j)\times 1$, $\mathrm{bdiag}_{N_j}(1_7 1'_7)$ is a block diagonal matrix with $1_7 1'_7$ replicated N_j times, and I_{7*N_j} is the identity matrix of size $(7 * N_j) \times (7 * N_j)$. As an example, if there were $N_j = 2$ prescribers in every clinic j, then $V_{j,(\sigma_Y,\rho,r)}$ is the following 14×14 matrix

$$\mathrm{cov}(Y_j) = \sigma_Y^2 \begin{bmatrix} \begin{bmatrix} 1 & r & \ldots & r \\ r & 1 & \ldots & r \\ \vdots & \vdots & \ddots & \vdots \\ r & r & & 1 \end{bmatrix} & \begin{bmatrix} \rho & \rho & \ldots & \rho \\ \rho & \rho & \ldots & \rho \\ \vdots & \vdots & \ddots & \vdots \\ \rho & \rho & & \rho \end{bmatrix} \\ \begin{bmatrix} \rho & \rho & \ldots & \rho \\ \rho & \rho & \ldots & \rho \\ \vdots & \vdots & \ddots & \vdots \\ \rho & \rho & & \rho \end{bmatrix} & \begin{bmatrix} 1 & r & \ldots & r \\ r & 1 & \ldots & r \\ \vdots & \vdots & \ddots & \vdots \\ r & r & & 1 \end{bmatrix} \end{bmatrix}.$$

Note that given this working covariance matrix: $\text{corr}(Y_{jit}, Y_{jit'}) = r$ for $t \neq t'$ is a "within-prescriber correlation coefficient" describing how the outcomes correlate over time for a prescriber i within a clinic j; and $\text{corr}(Y_{jit}, Y_{ji't'}) = \rho$ for $i \neq i'$ is a "inter-clinic correlation coefficient" describing how the outcomes between two prescribers within clinic j are correlated. For notational simplicity, let $\xi = (\sigma_Y, \rho, r)$ denote the variance components so that the working covariance matrix can be denoted by $V_{j,\xi}$.

The estimate $\hat{\theta}$ will be obtained as the solution to the estimating equations $\sum_j^J U_{j,\theta,\xi} = 0$ where $U_{j,\theta,\xi} = D_j' V_{j,\xi}^{-1} (Y_j - \mu_{j,\theta})$. Using a standard moments-based estimators for ξ based on the empirical residuals $Y_j - \hat{\mu}_{j,\hat{\theta}}$, we will iterate between estimates of ξ and estimates of θ until convergence.

We will estimate $\text{var}(\hat{\theta})$ using the following sandwich estimator, which provides valid inference even if the working covariance matrix is incorrect for the true $\text{var}(Y_j)$:

$$\hat{\text{var}}(\hat{\theta}) = \frac{1}{J} \hat{\Sigma}_B^{-1} \hat{\Sigma}_M \hat{\Sigma}_B^{-1}$$

where

$$\hat{\Sigma}_B = \frac{1}{J} \sum_j^J D_j' V_{j,\hat{\xi}}^{-1} D_j$$

and

$$\hat{\Sigma}_M = \frac{1}{J} \sum_j^J D_j' V_{j,\hat{\xi}}^{-1} B_{j,\hat{\xi}} (Y_j - \hat{\mu}_{j,\hat{\theta}})(Y_j - \hat{\mu}_{j,\hat{\theta}})' B_{j,\hat{\xi}} V_{j,\hat{\xi}}^{-1} D_j$$

where

$$B_{j,\hat{\xi}} = (I_{7*N_j} - D_j \hat{\Sigma}_B^{-1} D_j' V_{j,\hat{\xi}}^{-1})^{-1}.$$

The matrix B_j is used to provide a partial correction to small sample bias which may occur in the variance estimator due to small numbers of clinics.

4.4 Primary Hypothesis Test

The study's planned statistical test which is associated with the primary aim (and which we use to select the total study sample size, see Section (6) below) is based on a test of the null hypothesis that $\Delta = E_D(Y_{21} - Y_3) - E_A(Y_{21} - Y_3) = 0$. (Using the $t^* = t - 3$ transformation for time, the null hypothesis is $\Delta = E_D(Y_{18} - Y_0) - E_A(Y_{18} - Y_0) = 0$.)

This is a test of the null hypothesis that there is no average difference on change in MME from $t^* = 0$ to $t^* = 18$ (i.e., from $t = 3$ to $t = 21$) between implementation sequence D vs. implementation sequence A. Based on the pre-planned analysis model in display (2), $\Delta = 12\gamma_2 + 24\gamma_4 + 24\gamma_5$. Hence, the test that $H_0 : \Delta = 0$ corresponds to testing the null hypothesis that $H_0 : \gamma_2 + 12\gamma_4 + 12\gamma_5 = 0$.

To conduct this primary hypothesis test, we will calculate the sqrt-Wald statistic $\sqrt{W} = (\hat{\gamma}_2 + 12\hat{\gamma}_4 + 12\hat{\gamma}_5)/\sqrt{\hat{\text{var}}(\hat{\gamma}_2 + 12\hat{\gamma}_4 + 12\hat{\gamma}_5)}$ with the parameter and variance estimates calculated as described in subsection (4.3) above. We set the Type-I error rate to be $\alpha = 5\%$. Under the null, the sampling distribution of $\sqrt{W}$ is a standard normal distribution. However, to further protect against inflated Type I errors due to small numbers of clinics ($J = 44$), we will use the t-distribution in the Wald tests. Thus, we will reject the null hypothesis (in favor of the alternative hypothesis $H_A : \gamma_2 + 12\gamma_4 + 12\gamma_5 \neq 0$) if the value of $|\sqrt{W}| > t_{0.025,df=35} = 2.03$ (the $t_{\alpha/2}$-quantile with $J - 9 = 44 - 9 = 35$ degrees of freedom). If, instead, $|\sqrt{W}| \leq 2.03$, we will state that "based on the planned, primary hypothesis test, there is no evidence to suggest that there is an average difference on change in MME from $t^* = 0$ to $t^* = 18$ (i.e., from $t = 3$ to $t = 21$) between implementation sequence D vs. implementation sequence A." Note that this does not mean that there is no difference between implementation sequence D vs. implementation sequence A.

4.5 Reporting Results

In the primary aim manuscript, we will make a clear distinction between the study's single hypothesis test and other data analyses/contrasts:

- First, we will report results of the study's primary aim hypothesis test, as described in Section 4.4 above. This includes reporting the p-value for the study's single primary hypothesis.
- Second, following the presentation of results for the study's primary hypothesis test, we will report estimates and 95% confidence intervals for each of the γ parameters, as described above, as well as for other interesting associations or effects (e.g., other pairwise comparisons between the implementation sequences). This includes reporting the results of any other additional stability analyses (e.g., models that do not assume linearity over time in the marginal mean, or results that examine other ways to address missingness). No p-values are reported for any of these additional analyses.

5 Missing Data

The covariates X and the repeated measures outcomes $Y_{j,i,t}$ used in the primary aim analysis are to be collected based on electronic health record (EHR) data pulls. There can be no missing data for the covariates X and the baseline outcome $Y_{i,j,3}$ since these measures are used to stratify the J clinics that make up the intent-to-treat sample immediately prior to the first randomization. That is, a clinic does not enter the intent-to-treat sample unless it is randomized, but a clinic cannot be randomized unless its values for X and $Y_{i,j,3}$ are known. Thus, for all clinics in this trial, we will have at least the following data observed: $(X, Y_{i,j,3}, A_1, A_2)$. Further, we expect it to be rare to have any missing data in the remaining 18 outcomes $Y_{j,i,t}$ $(t > 3)$. Nevertheless, possible reasons for missingness in these outcomes may be due to clinician turnover (without ability to obtain their outcomes, subsequently) or possible errors in the EHR system that lead to data loss.

In case of missing data for $Y_{j,i,t}$ $(t > 3)$: Prior to the analysis described above, a thorough investigation of reasons for missing data and mechanisms for missing data will be carried out to understand patterns and key predictors of missingness. Using this information, missing data will be multiply imputed (MI) using chained-equations. The primary aim analysis and hypothesis test described above will be carried out for each of the multiply imputed data sets (≥ 50 imputed data sets); and the estimates and tests pooled across the multiple imputations using Rubin's rules. In additional stability analyses, the analysis will also be conducted without the MI data (using only complete cases). The pre-planned primary aim analysis and test, as described above, will be based on the multiply-imputed data; however, any discrepancies in the analysis with and without the MI data will be carefully examined and reported.

6 Sample Size Calculation

The total sample size for this study is based on the primary aim: a comparison on average difference on change in MME from $t^* = 0$ to $t^* = 18$ (i.e., from $t = 3$ to $t = 21$) between implementation sequence D vs. implementation sequence A. This is a comparison between two of the four groups embedded in the trial (see Table 1). The sample size calculator for this comparison is a straightforward adjustment to the sample size calculator for a standard two-sample hypothesis test. The adjustment accounts for the clustering of prescribers within clinics through a variance inflation factor (VIF) of $1 + (m - 1)\rho$, where m is the average number of prescribers per clinic and ρ is inter-clinic correlation coefficient (ICC) for MME at month $t = 21$ $(t^* = 18)$. Based on implementation clinics in the R34 pilot data, the ICC was estimated to be $\rho = 0.14$. Assuming an average of $m = 6$ prescribers per clinic (based on information from the new health systems that have agreed to participate), a Type-1 error rate of $\alpha = 5\%$, and $\rho = 0.14$, a minimum of 64 prescribers in each group (or 11 clinics per group) will provide at least 80% power to detect a moderate effect size of

$d = 2/3$ between the two implementation sequences on change in MME. Because we have four groups in this trial, the minimum total study sample size is $N = 256$ clinician prescribers, corresponding to $J = 44$ clinics.

Based on the pilot data that found a standard deviation of 35 for MME, an effect size of $d = 2/3$ corresponds to detecting an average difference of at least 23 on the MME between the two implementation sequences after 21 months. The above calculation is expected to be conservative because it does not account for within-prescriber correlation in MME, but which is accomodated for in the longitudinal analyses and could permit detection of smaller differences in MME.

7 Additional Study Analyses

The goal of this supplement is to describe the primary aim analyses (and the corresponding primary aim hypothesis test), which are to understand how the sequences of implementation strategies impact the precsribing practices of prescribers within clinics. Consistent with this primary aim, the study's primary outcome is at the prescriber-level.

However, the study does collect additional data, including data on other patient-level and clinic-level outcomes. Additional analyses, not described here, will involve understanding how the sequences of implementation strategies impact outcomes at different levels. For example, additional analyses might involve understanding how the sequences of implementation impact outcomes at the patient-level. Or, additional analyses might focus on the impact of the sequences of implementation strategies on outcomes at the clinic-level such as cost or other policies enacted at the clinic-level.

Further, this supplement does not describe the analysis protocol for exploratory aims, such as the investigation of baseline and time-varying moderators of the effect of the sequences of implementation strategies.
