# Supplementary material for: The Balanced Opioid Initiative: protocol for a clustered, sequential, multiple-assignment randomized trial to construct an adaptive implementation strategy to improve guideline-concordant opioid prescribing in primary care
Source: Implement Sci. 2020 Apr 25;15:26. doi: 10.1186/s13012-020-00990-4 (PMC7183389; doi:10.1186/s13012-020-00990-4)
Supplement: Supplementary file 4 — Additional file 4:. Proof of ethics [file 13012_2020_990_MOESM4_ESM.pdf]

Minimal Risk IRB (Health Sciences)
1/28/2020

**Submission ID number:**	2018-1276-CP008
**Title:**	Promoting the implementation of clinical guidelines for opioid prescribing in primary care using systems consultation
**Principal Investigator:**	ANDREW R QUANBECK
**Point-of-Contact:**	NICHOLAS DONEL SCHUMACHER
**IRB Staff Reviewer:**	ANNA MARTIN

A designated MR IRB member conducted an expedited review of the above-referenced change of protocol application. The change of protocol application was approved by the IRB member. The change of protocol application qualified for expedited review pursuant to 45 CFR 46.110 and, if applicable, 21 CFR 56.110 and 38 CFR 16.110. You must log in to your ARROW account in order to view the specific changes approved by the IRB.

To access the materials approved by the IRB, including any stamped consent forms, recruitment materials and the approved protocol, if applicable, please log in to your ARROW account and view the documents tab in the submission's workspace.

If you requested a HIPAA waiver of authorization, altered authorization and/or partial authorization, please log in to your ARROW account and view the history tab in the submission's workspace for approval details.

You have identified the following financial sources to support the research activities in this IRB application:

- QUANBECK, ANDREW R - Promoting the implementation of clinical guidelines for opioid prescribing in primary care using systems consultation - DHHS, PHS, NATIONAL INSTITUTES OF HEALTH - 1 R01 DA047279-01

If this information is incorrect, please submit a change to modify your application as appropriate.

Please review the Investigator Responsibilities guidance (https://kb.wisc.edu/hsirbs/page.php?id=18881) which includes a description of IRB requirements for submitting continuing review progress reports, changes of protocol and reportable events.

If you have general questions, please contact the Health Sciences IRBs at 608-263-2362. For

questions related to this submission, contact the assigned staff reviewer.
