# Supplementary material for: The Balanced Opioid Initiative: protocol for a clustered, sequential, multiple-assignment randomized trial to construct an adaptive implementation strategy to improve guideline-concordant opioid prescribing in primary care
Source: Implement Sci. 2020 Apr 25;15:26. doi: 10.1186/s13012-020-00990-4 (PMC7183389; doi:10.1186/s13012-020-00990-4)
Supplement: Supplementary file 5 — Additional file 5:. Proof of funding [file 13012_2020_990_MOESM5_ESM.pdf]

RESEARCH
Department of Health and Human Services
National Institutes of Health

NATIONAL INSTITUTE ON DRUG ABUSE

Federal Award Date: 06/10/2019

Grant Number: 5R01DA047279-02
FAIN: R01DA047279

Principal Investigator(s):
Andrew Quanbeck, PHD

Project Title: Promoting the implementation of clinical guidelines for opioid prescribing in primary care using systems consultation

MELTZER, DEBORAH M
Assistant Dean
750 HIGHLAND AVE
4115 HLTH SCI LEARNING CTR
MADISON, WI 537052221

Award e-mailed to: NIH@rsp.wisc.edu

Period Of Performance:
Budget Period: 07/01/2019 – 06/30/2020
Project Period: 09/30/2018 – 06/30/2023

Dear Business Official:

The National Institutes of Health hereby awards a grant in the amount of $726,877 (see “Award Calculation” in Section I and “Terms and Conditions” in Section III) to UNIVERSITY OF WISCONSIN-MADISON in support of the above referenced project. This award is pursuant to the authority of 42 USC 241 42 CFR 52 and is subject to the requirements of this statute and regulation and of other referenced, incorporated or attached terms and conditions.

Acceptance of this award including the “Terms and Conditions” is acknowledged by the grantee when funds are drawn down or otherwise obtained from the grant payment system.

Each publication, press release, or other document about research supported by an NIH award must include an acknowledgment of NIH award support and a disclaimer such as “Research reported in this publication was supported by the National Institute On Drug Abuse of the National Institutes of Health under Award Number R01DA047279. The content is solely the responsibility of the authors and does not necessarily represent the official views of the National Institutes of Health.” Prior to issuing a press release concerning the outcome of this research, please notify the NIH awarding IC in advance to allow for coordination.

Award recipients must promote objectivity in research by establishing standards that provide a reasonable expectation that the design, conduct and reporting of research funded under NIH awards will be free from bias resulting from an Investigator’s Financial Conflict of Interest (FCOI), in accordance with the 2011 revised regulation at 42 CFR Part 50 Subpart F. The Institution shall submit all FCOI reports to the NIH through the eRA Commons FCOI Module. The regulation does not apply to Phase I Small Business Innovative Research (SBIR) and Small Business Technology Transfer (STTR) awards. Consult the NIH website http://grants.nih.gov/grants/policy/coi/ for a link to the regulation and additional important information.

If you have any questions about this award, please contact the individual(s) referenced in Section IV.

Sincerely yours,

EDITH L. DAVIS
Grants Management Officer
NATIONAL INSTITUTE ON DRUG ABUSE

Additional information follows

SECTION I – AWARD DATA – 5R01DA047279-02

Award Calculation (U.S. Dollars)

**Salaries and Wages**	$216,783
**Fringe Benefits**	$75,875
**Personnel Costs (Subtotal)**	$292,658
**Consultant Services**	$25,945
**Materials & Supplies**	$7,315
**Travel**	$3,599
**Other**	$61,750
**Subawards/Consortium/Contractual Costs**	$128,238

**Federal Direct Costs**	$519,505
**Federal F&A Costs**	$207,372
**Approved Budget**	$726,877
**Total Amount of Federal Funds Obligated (Federal Share)**	$726,877
**TOTAL FEDERAL AWARD AMOUNT**	$726,877

**AMOUNT OF THIS ACTION (FEDERAL SHARE)**	$726,877

SUMMARY TOTALS FOR ALL YEARS		
**YR**	**THIS AWARD**	**CUMULATIVE TOTALS**
2	$726,877	$726,877
3	$721,075	$721,075
4	$717,148	$717,148
5	$692,720	$692,720

Recommended future year total cost support, subject to the availability of funds and satisfactory progress of the project

Fiscal Information:
CFDA Name: Drug Abuse and Addiction Research Programs
CFDA Number: 93.279
EIN: 1396006492A1
Document Number: RDA047279A
PMS Account Type: P (Subaccount)
Fiscal Year: 2019

IC	CAN	2019	2020	2021	2022
DA	8472628	$726,877	$721,075	$717,148	$692,720

Recommended future year total cost support, subject to the availability of funds and satisfactory progress of the project

NIH Administrative Data:
PCC: CV/ACA / **OC**: 414E / **Released**: PFLEMING 06/07/2019
Award Processed: 06/10/2019 12:11:26 AM

SECTION II – PAYMENT/HOTLINE INFORMATION – 5R01DA047279-02

For payment and HHS Office of Inspector General Hotline information, see the NIH Home Page at http://grants.nih.gov/grants/policy/awardconditions.htm

SECTION III – TERMS AND CONDITIONS – 5R01DA047279-02

This award is based on the application submitted to, and as approved by, NIH on the above-titled project and is subject to the terms and conditions incorporated either directly or by reference in the following:

a. The grant program legislation and program regulation cited in this Notice of Award.
b. Conditions on activities and expenditure of funds in other statutory requirements, such as those included in appropriations acts.

c. 45 CFR Part 75.
d. National Policy Requirements and all other requirements described in the NIH Grants Policy Statement, including addenda in effect as of the beginning date of the budget period.
e. Federal Award Performance Goals: As required by the periodic report in the RPPR or in the final progress report when applicable.
f. This award notice, INCLUDING THE TERMS AND CONDITIONS CITED BELOW.

(See NIH Home Page at http://grants.nih.gov/grants/policy/awardconditions.htm for certain references cited above.)

Research and Development (R&D): All awards issued by the National Institutes of Health (NIH) meet the definition of "Research and Development" at 45 CFR Part§ 75.2**.** As such, auditees should identify NIH awards as part of the R&D cluster on the Schedule of Expenditures of Federal Awards (SEFA). The auditor should test NIH awards for compliance as instructed in Part V, Clusters of Programs. NIH recognizes that some awards may have another classification for purposes of indirect costs. The auditor is not required to report the disconnect (i.e., the award is classified as R&D for Federal Audit Requirement purposes but non-research for indirect cost rate purposes), unless the auditee is charging indirect costs at a rate other than the rate(s) specified in the award document(s).

This institution is a signatory to the Federal Demonstration Partnership (FDP) Phase VI Agreement which requires active institutional participation in new or ongoing FDP demonstrations and pilots.

An unobligated balance may be carried over into the next budget period without Grants Management Officer prior approval.

This grant is subject to Streamlined Noncompeting Award Procedures (SNAP).

This award is subject to the requirements of 2 CFR Part 25 for institutions to receive a Dun & Bradstreet Universal Numbering System (DUNS) number and maintain an active registration in the System for Award Management (SAM). Should a consortium/subaward be issued under this award, a DUNS requirement must be included. See http://grants.nih.gov/grants/policy/awardconditions.htm for the full NIH award term implementing this requirement and other additional information.

This award has been assigned the Federal Award Identification Number (FAIN) R01DA047279. Recipients must document the assigned FAIN on each consortium/subaward issued under this award.

Based on the project period start date of this project, this award is likely subject to the Transparency Act subaward and executive compensation reporting requirement of 2 CFR Part 170. There are conditions that may exclude this award; see http://grants.nih.gov/grants/policy/awardconditions.htm for additional award applicability information.

In accordance with P.L. 110-161, compliance with the NIH Public Access Policy is now mandatory. For more information, see NOT-OD-08-033 and the Public Access website: http://publicaccess.nih.gov/.

This award provides support for one or more clinical trials. By law (Title VIII, Section 801 of Public Law 110-85), the "responsible party" must register "applicable clinical trials" on the ClinicalTrials.gov Protocol Registration System Information Website. NIH encourages registration of all trials whether required under the law or not. For more information, see http://grants.nih.gov/ClinicalTrials_fdaaa/

This award provides support for one or more NIH defined Phase III Clinical Trials. The NIH Policy for research supported as an NIH Phase III Clinical Trial has been amended in Section II.B. of the NIH Guidelines on the Inclusion of Women and Minorities as Subjects in Clinical Research –

Amended October 2001 (see
http://grants.nih.gov/grants/funding/women_min/guidelines_amended_10_2001.htm).

A description of plans to conduct analyses, as appropriate, by sex/gender and racial/ethnic groups must be included in clinical trial protocols. Cumulative subject accrual and progress in conducting subset analyses must be reported to NIH in the annual Progress Reports. Final analyses of sex/gender and racial/ethnic differences must be reported in the required Final Progress Report or Competitive Renewal Applications (or Contract Renewals/Extensions) as stated in Section II.B. of the Guidelines.

In accordance with the regulatory requirements provided at 45 CFR 75.113 and Appendix XII to 45 CFR Part 75, recipients that have currently active Federal grants, cooperative agreements, and procurement contracts with cumulative total value greater than $10,000,000 must report and maintain information in the System for Award Management (SAM) about civil, criminal, and administrative proceedings in connection with the award or performance of a Federal award that reached final disposition within the most recent five-year period. The recipient must also make semiannual disclosures regarding such proceedings. Proceedings information will be made publicly available in the designated integrity and performance system (currently the Federal Awardee Performance and Integrity Information System (FAPIIS)). Full reporting requirements and procedures are found in Appendix XII to 45 CFR Part 75. This term does not apply to NIH fellowships.

Treatment of Program Income:
Additional Costs

SECTION IV – DA Special Terms and Conditions – 5R01DA047279-02

Clinical Trial Indicator: Yes
This award supports one or more NIH-defined Clinical Trials. See the NIH Grants Policy Statement Section 1.2 for NIH definition of Clinical Trial.

DATA AND SAFETY MONITORING

This award is subject to the current Data and Safety Monitoring Plan (DSMP) submitted and previously approved by NIDA. Any changes in the DSMP must be reviewed and approved by the Program Official. If changes are approved, the approval will be reflected on the Notice of Award. If changes are not approved, the Principal Investigator must revise the DSMP to the satisfaction of the Program Official. The Principal Investigator must provide a DSMP for any new trial that is to be conducted under this grant.

CLINICAL TRIAL(S) DISSEMINATION PLAN

The clinical trial(s) supported by this award is subject to the plan dated 9/07/18 submitted to NIH and the NIH policy on Dissemination of NIH-Funded Clinical Trial Information. The plan states that the clinical trial(s) funded by this award will be registered in ClinicalTrials.gov not later than 21 calendar days after enrollment of the first participant and primary summary results reported in ClinicalTrials.gov, not later than one year after the completion date. The reporting of summary results is required by this term of award even if the primary completion date occurs after the period of performance.

This award is subject to additional certification requirements with each submission of the Annual, Interim, and Final Research Performance Progress Report (RPPR). The recipient must agree to the following annual certification when submitting each RPPR. By submitting the RPPR, the AOR signifies compliance, as follows:

In submitting this RPPR, the SO (or PD/PI with delegated authority), certifies to the best of his/her knowledge that, for all clinical trials funded under this NIH award, the recipient and all investigators conducting NIH-funded clinical trials are in compliance with the recipient's plan addressing compliance with the NIH Policy on Dissemination of NIH-Funded Clinical Trial Information. Any clinical trial funded in whole or in part under this award has been registered in ClinicalTrials.gov or will be registered not later than 21 calendar days after enrollment of the first participant. Summary results have been submitted to ClinicalTrials.gov or will be submitted not later than one year after the completion date, even if the completion date occurs after the period of performance.

FOA REQUIREMENTS
This award is subject to the requirements detailed in **PAR18-007**, entitled, Dissemination and Implementation Research in Health (R01 Clinical Trial Optional), released on 11/3/17**,** which is incorporated by reference as terms and conditions of this award.

Copies of this announcement may be accessed at the following URL:
http://grants.nih.gov/grants/guide/pa-files/PAR-18-007.html

NIDA TERMS
In conjunction with the Acknowledgment of Federal Funding Requirement (as specified in the NIH Grants Policy Statement, Appropriation Mandates- http://grants.nih.gov/policy/nihgps/index.htm), in order to most effectively disseminate research results, advance notice should be given to NIDA that research findings are about to be published so that we may coordinate accurate and timely release to the media. This information will be embargoed until the publication date. Any press notification should be coordinated with the NIDA Press Officer who can be reached at (301) 443-6245.

The National Institute on Drug Abuse (NIDA) encourages data harmonization to increase comparability, collaboration, and scientific yield of research on drug abuse. Towards that end, NIDA strongly encourages human-subject studies to incorporate a series of measures from the Substance Abuse and Addiction Core and Specialty collections, which are available in the PhenX Toolkit at http://www.phenxtoolkit.org. For more information about NIDA's data harmonization efforts, please see NOT-DA-12-008 at http://grants.nih.gov/grants/guide/notice-files/NOT-DA-12-008.html.

STAFF CONTACTS

The Grants Management Specialist is responsible for the negotiation, award and administration of this project and for interpretation of Grants Administration policies and provisions. The Program Official is responsible for the scientific, programmatic and technical aspects of this project. These individuals work together in overall project administration. Prior approval requests (signed by an Authorized Organizational Representative) should be submitted in writing to the Grants Management Specialist. Requests may be made via e-mail.

Grants Management Specialist: Edith L. Davis
Email: edavis1@nida.nih.gov **Phone**: 301-827-6697

Program Official: Aria Crump
Email: acrump@nida.nih.gov **Phone**: 301-443-6504 **Fax**: 301-480-2542

SPREADSHEET SUMMARY
GRANT NUMBER: 5R01DA047279-02

INSTITUTION: UNIVERSITY OF WISCONSIN-MADISON

Budget	Year 2	Year 3	Year 4	Year 5
Salaries and Wages	$216,783	$224,165	$221,344	$216,593
Fringe Benefits	$75,875	$78,458	$77,471	$75,808
Personnel Costs (Subtotal)	$292,658	$302,623	$298,815	$292,401
Consultant Services	$25,945	$6,572	$2,240	$7,888
Materials & Supplies	$7,315			
Travel	$3,599		$5,273	$5,273
Other	$61,750	$76,950	$54,150	$38,950
Subawards/Consortium/Contractual Costs	$128,238	$128,238	$161,838	$161,838
Publication Costs		$1,330	$2,470	$2,470
TOTAL FEDERAL DC	$519,505	$515,713	$524,786	$508,820
TOTAL FEDERAL F&A	$207,372	$205,362	$192,362	$183,900
TOTAL COST	$726,877	$721,075	$717,148	$692,720

Facilities and Administrative Costs	Year 2	Year 3	Year 4	Year 5
F&A Cost Rate 1	53%	53%	53%	53%
F&A Cost Base 1	$391,267	$387,475	$362,948	$346,982
F&A Costs 1	$207,372	$205,362	$192,362	$183,900
